# Engaging Students in Advancing Campus Tobacco-Free Policies: A Qualitative Study of California Community Colleges’ Efforts

**DOI:** 10.5888/pcd20.230082

**Published:** 2023-11-09

**Authors:** Setareh Harsamizadeh Tehrani, Sang Leng Trieu, Lien Dao, Carissa Samuel, Camillia K. Lui

**Affiliations:** 1 Student Health Center, Ohlone College, Fremont, California; 2 Alcohol Research Group, Public Health Institute, Emeryville, California

## Abstract

**Introduction:**

Tobacco use remains a serious problem for young adults. Given the large number of young adults attending college, a tobacco-free campus is one strategy to reduce tobacco use. Young adult engagement is recognized as a common strategic practice in tobacco control policy efforts, especially in changing social norms around tobacco use. Community colleges can leverage and engage students in adoption of campus 100% tobacco-free policies. This qualitative study examines the importance of student engagement in advancing 100% tobacco-free policies in community colleges and identifies strategies for campuses to involve students in such efforts.

**Methods:**

We selected 12 community colleges and conducted key informant interviews with campus and community-based organizations that were involved in campus policy adoption efforts. We conducted 33 semistructured interviews and transcribed, coded, and analyzed them by using a thematic analytic framework with inductive and deductive approaches to examine student engagement processes.

**Results:**

Community colleges represented campuses with (n = 6) and without (n = 6) tobacco-free policy and varied by geography (urban vs rural) and student population size. Three main themes emerged: 1) no “wrong door” for students to engage in tobacco control work, 2) a myriad of ways for students to be involved in policy adoption, and 3) benefits of student engagement.

**Conclusion:**

We found that students are doers, allies, and champions in adoption of 100% campus tobacco-free policy. Colleges should leverage their campuses’ most important assets — students — to be agents of change and to involve them in the full spectrum of interventions and advocacy.

**Summary**

**What is known on this topic?**

In 2021, young US adults had the highest smoking and vaping rates, and smoking prevalence is higher among community college students compared with their 4-year counterparts.

**What is added by this report?**

Student engagement is recognized as a key strategy in tobacco control policy efforts. However, research on community colleges and on student engagement in policy efforts is limited. This qualitative study describes the levels, roles, and value of engaging students in advancing a 100% tobacco-free policy in California community colleges.

**What are the implications for public health practice?**

Colleges should leverage their campuses’ most important assets — students — as agents of change and involve them in the full spectrum of tobacco control efforts.

## Introduction

In 2021, young US adults aged 18 to 25 years had the highest smoking and vaping rate (14.1% or 4.7 million people) (1). Given the number of young adults attending college, a tobacco-free campus is one strategy to reduce tobacco use through student engagement (2). California Community Colleges (CCCs), the nation’s largest higher education system, passed a 2018 resolution supporting the adoption and implementation of 100% tobacco-free policies (TFPs). Because the resolution is not binding, only 66% of CCCs are completely tobacco-free as of 2023 (3).

As more young adults pursue higher education (4), colleges are an opportune environment for tobacco prevention and cessation efforts. However, much of the research has focused on 4-year colleges, leaving community colleges an understudied population (5–8), which is surprising given that community college students make up more than two-fifths (42%) of all US undergraduates (9). Smoking prevalence, particularly daily smoking, is higher among community college students compared with their 4-year counterparts, and community college students are less likely to quit (10–12). Similarly, student engagement in community colleges differs from that in 4-year universities due to several factors. Community colleges tend to enroll a more diverse student body than 4-year universities, with higher proportions of low-income and first-generation college students (5). Additionally, community colleges often have fewer support services than 4-year universities (13), and the transitional nature of a community college along with a shared governance structure make TFP-related student engagement more complicated.

For more than 3 decades, student engagement has been recognized as a strategic practice in tobacco policy efforts (14). Student involvement can advance comprehensive tobacco control efforts through social norm change, particularly with counter-marketing efforts. Student engagement can yield high economic returns at low cost. The American College Health Association’s (ACHA) Position Statement on Tobacco on College and University Campuses recommends the development of a tobacco task force with student involvement (15). The literature on college students’ involvement in tobacco control efforts is limited. After passing a TFP, one campus found that student ambassadors improved compliance and reduced cigarette butts at campus hotspots (16). Given the dearth of research on student involvement in campus policy efforts, we conducted a qualitative study to examine the importance of student engagement in advancing 100% TFPs in community colleges and identify strategies for campuses to involve students in such efforts.

## Methods

In this phenomenological study, 12 community colleges were purposively selected on the basis of criteria from our parent study that focused on facilitators and barriers to college TFP adoption. Selection criteria included geographic location and policy status and was informed by prior study results (17,18). Up to 3 key informants at each community college were recruited on the basis of their knowledge of or direct experience with the TFP adoption process and included students, staff, faculty, college leaders, or people employed with a tobacco-related community-based organization or public health department. Key informants were recruited through our study advisory board (including the California Youth Advocacy Network and the Health Services Association California Community Colleges), websites, and referrals from key informants. Recruitment was done via email and telephone. A total of 33 key informants participated.

A semistructured interview guide was developed using Ickes and colleagues’ Campus Assessment of Readiness to End Smoking (19) (including resources, leadership, knowledge, campus climate, political climate, and existing tobacco control policies) and Frohlich and Abel’s Institutional Study of Inequalities in Smoking (ISIS) framework (20) (including individual efforts and collective networks). Questions included experience working at the community college or in the tobacco control field, knowledge or insights on the policy adoption process, and key players, including students. Virtual interviews were conducted from January 2021 through January 2022. All key informants provided informed consent and permission to record the interview. The Public Health Institute’s Human Subjects Review Committee provided institutional board review approval (study exemption no. I18–015a).

We followed Braun and Clark’s reflexive thematic analytic framework, in which we acknowledged our positionality that reflects our own experiences (as students, a college administrator, and an external community partner) and our role as researchers in the interpretations of the participants’ experiences (21,22). Based on Ickes and colleagues’ campus readiness assessment and ISIS framework (19,20), a codebook was developed deductively (eg, campus leadership, student engagement) and, after review of the first 6 interviews as a group, inductively as new concepts emerged. The coding process began as a group with the first 3 transcripts to ensure consistency with interpretation of codes. Subsequent transcripts involved 2 coding teams (2 community college–level research assistants with support from S.L.T. and 1 graduate-level research assistant with support from C.K.L.) who independently applied codes again for the first 3 transcripts. When coding discrepancies occurred, the team discussed the issues, came to a consensus on code definition, and documented the resolution in the codebook, which was then applied to the remaining transcripts to ensure consistency. The coding teams independently coded the remaining interviews. Weekly coding sessions were conducted, and questions or conflicts were discussed and resolved. Dedoose software was used for coding (23). Excerpts under the “student” code were extracted for this study and entered into Microsoft Excel to identify patterns (Microsoft Corporation). After first review of the 132 excerpts, 10 potential themes emerged. After the second review, we prioritized 3 themes based on the study goals to highlight unique aspects of the community college experience and inform student engagement in policy adoption. Through group discussion, exemplar quotes were selected to best characterize each theme.

## Results

The selected community colleges differed by rural and urban geography and student population size. Key informants provided unique perspectives of community college students, given their relationship as students themselves or the fact that they worked closely with students through campus services or policy efforts (Table 1). Eight colleges actively involved students in the policy adoption efforts, and among them, 6 colleges or community organizations paid students via stipend or employment. Three key themes and corresponding exemplar quotes are presented (Table 2).

**Table 1:** Characteristics of Colleges (N = 12) and Key Informants (N = 33) in Study Sample, Survey on Tobacco-Free Policies of California Community Colleges, 2021–2022

College no.	Region, geography	Has 100% tobacco-free policy, year policy adopted	Student population size, 2019	Campus lead in policy adoption	Has external campus partner	Student involvement in policy efforts	Key informant no., title
CS1	Northern California, rural	Yes, 2019	9,315	Student health center and student services	No	Yes	17, Student health center director 19, Student health center director 20, Student services director
CS2	Northern California, rural	No	10,942	Campus smoke-free task force	CBO	No	14, CBO project director 16, CBO health educator 33, Student health center director
CS3	Bay Area, urban	Yes, 2018	24,344	Campus–community smoke-free task force	CBO	No	2, Student health center director 3, CBO project director 4, Student health services staff
CS4	Bay Area, suburb	Yes, 2021^a^	8,537	Faculty–community organization	CBO	Yes, paid	5, College faculty 6, Student health center nurse 7, Student 13, CBO project director/staff
CS5	Central California, urban	Yes, 2016	11,840	Campus–community task force	County public health department	Yes	25, Student health center nurse 26, College vice president 34, County tobacco control specialist
CS6	Central California, urban	No	13,856	Student health center	No	No	24, Student health center director
CS7	Los Angeles, urban	Yes, 2013	29,057	Student health center and student services	No	No	29, College institutional effectiveness director 35, College vice president
CS8	Los Angeles, urban	No	19,997	Student health center	CBO	Yes, paid	11, Student health center nurse 12, Student health center director 18, CBO project director
CS9	Southern California, urban	No	16,405	Student health center	County public health department	Yes, paid	23, Student health center director 37, County tobacco control program supervisor
CS10	Southern California, urban	No	14,228	Student group–community organization	CBO	Yes, paid	27, CBO senior tobacco control manager 32, CBO community engagement manager 36, Student
CS11	Northern California, rural	Yes, 2021	1,862	Student services	CBO	Yes, paid	15, Student/CBO college coordinator 21, CBO project director 31, College vice president
CS12	Central California, rural	No	2,873	Student health center	CBO	Yes, paid	9, CBO project director 10, Student health center director 22, College director of research

Abbreviation: CBO, community-based organization.

a At the start of the study CS4 did not have a tobacco-free policy but adopted the policy during this study.

**Table 2:** List of Exemplar Quotes From Key Themes, Survey on Tobacco-Free Policies of California Community Colleges, 2021–2022

Quote no.	Quote code	Theme	Quote
1	CS11, no. 15 student and external partner organization	Theme 1: no wrong door for student engagement in tobacco efforts	“Our premed and nursing clubs would have been probably the ones off the top of our head.”
2	CS12, no. 9 external partner organization		“When they started bringing that topic [campus smoke-/tobacco-free policy] to the associated students, the feeling among the students was that they were generally supportive. There was no student who was like ‘No, we don’t want this to happen,’ they were all like ‘Yeah, that makes sense. We should do this.’”
3	CS11, no. 15 student and external partner organization		“We found that students do not have to come from a specific background to join tobacco policy efforts. They could be in any academic field, even athletics since ‘the teams are big so like if you get one team involved, you can easily get 10 to 30 people out of it. . . . With . . . Earth Day . . . we have at least 15 basketball players choose themselves. . . . If you get one person on the team excited about it, then we’re likely [to have] . . . a whole bunch of fans [too].’”
4	CS2, no. 16 external partner organization		“We can have those points of contact where we say like, ‘Hey, you were on student senate, we heard that you were interested in this, come join our advisory committee,’ and then we’re able to build up those ranks of people on campus who do have the passion, interests, and also have been in a leadership role that like faculty leadership would respond to on campus.”
5	CS7, no. 35 college administrator		“The peer health educators . . . were doing a campaign associated with what [e-cigarette] and vaping could do, like mouth cancer. . . . They were trying to bring some awareness about that and how e-cig smoke actually can do worse damage to the lung.”
6	CS12, no. 9 external partner organization		“[A strategy that has been working for us is] paid student internships. I think bringing that social justice and environmental justice to [the] lens of student interns so that they get kind of passionate about [tobacco-free policy] has been helpful.”
7	CS12, no. 22 college administrator		“[An external partner] had employed two of our students as interns, and my motivation was to provide an educational opportunity for those students. . . . They were really driving.”
8	CS2, no. 16 external partner organization	Theme 2: myriad levels of student engagement in tobacco-policy work	“It goes back to that ownership of what’s happening on campus, and then it’s working with those students to do different evaluations, or things on campus, continuing to raise awareness, setting up meetings, usually with the associated students or the student senate, whatever the structure is on campus.”
9	CS9, no. 37 external partner organization		“Students created their own artwork depicting why they thought that the campuses should go smoke-free. . . . Student artwork made it onto a bus shelter, ads, and billboards and other artwork . . . was placed on and around the school campuses.”
10	CS9, no. 37 external partner organization		“[The students] created this really wonderful kind video that shows testimonials from different students and faculty sharing why they wanna see their campuses go smoke-free.”
11	CS5, no. 26 college administrator		“[If students] wanted to get in front of the board and say why this shouldn’t happen that could have made it a much more difficult process to adopt the policy, but you know, thankfully for us, we had a student body that again understood that this was the right thing and they were supportive and helped us implement as opposed to trying to be obstructionist at all.”
12	CS9, no. 37 external partner organization-LLA		“[We have been] gauging the students as necessary, but then you have to be able to tell them, Ok, these are the steps that we need to take. So yes, gather the data, gather the evidence, show the support from the students.”
13	CS10, no. 32 external partner organization		“Some of the students from the school actually came out and spoke in city council, and so they’ve tried to also make sure that the students are also involved in local [city] policy, not just at their school. And they really enjoyed it.”
14	CS12, no. 9 external partner organization		“[Students] did really advocate for the policy. They did this survey; I know they did presentations to decision-making groups. I think they went to the faculty senate and the staff; they might have talked to the president and the students, and they were trying to gain support from all these decision-making bodies.”
15	CS1, no. 20 college administrator	Theme 3: benefits of student engagement	“I very much looked to students just for their experience, and perspective. . . . And so I think [it’s] so important . . . to put students in . . . a position of power. You know kind of let them take a lead, and not only does that obviously give them great experience that they’ll take later in life, but I feel like I learn so much from students.”
16	CS12, no. 10 student health center		“I know the main players that are looked to for campus policies are students. So if students initially say that’s what they want, they can rally around the committee structure that moves it up into policy.”
17	CS10, no. 36 student		“It has to be a community effort because if I could get 75% or 52% of the students to say that this is important and this is something that they value in their college community, or even probably 35% or you know what whatever the statistic could be, then it would become important to the board and it would become important to the people that oversee the bigger policies.”
18	CS12, no. 9 external partner organization		“Because if we didn’t have Jon [student intern], the students wouldn’t have adopted this resolution [in student government] I don’t think. And Jon wouldn’t have known that this is such an important issue unless we advertised a paid student internship.”
19	CS5, no. 26 college administrator		“You know we did have students at everywhere along the way weighing in, and I think they did a good job representing what the students wanted the campus to look like.”
20	CS12, no. 10 student health center		“The students really picked up that piece saying that you’re not free you know, it’s not a freedom issue to make other people sick . . . and I think it was best to come from the students.”
21	CS1, no. 20 college administrator		“So, while there was you know obviously a lot of people feeling alienated and upset about the policy, there were also those students who could see the value in it, and I felt like he was such an asset to trying to reach out to those students and help them understand like we really just want what’s best for you. We’re not trying to alienate you from this campus, this campus is just as much yours as the rest of ours.”
22	CS10, no. 27 external partner organization		“What really got them [the students] involved . . . was just all the policy work that we were doing and the opportunities for them to be part of what [the American Cancer Society] could offer, [whether] it will be state work or going to DC . . . as part of our national lobby day effort. Or to get involved with the larger effort because a lot of them were looking to transfer to a 4-year university so that appealed to them.”
23	CS12, no. 9 external partner organization		“Yeah, he [student intern] kind of cared about tobacco and smoking, but it’s probably not his top issue that he cares about. But bringing him into this and then having him host and attend different webinars and he’s just like really gotten into it and really like this social justice part of it, inequity and stuff. And so now he can take that passion with him.”

Abbreviation: LLA, local lead agency.

### Theme 1: No “wrong door” for student engagement in tobacco efforts

The first theme emphasized that there is no “wrong door” for community college students to get involved in TFP work, with many opportunities for students to participate in committees advocating for TFP. Key informants reported that most students got involved formally through campus organizations such as student government (eg, Associated Students, student senate), student clubs, and health care–related majors. For example, one informant considered recruiting students mainly from health-related majors (Table 2, quote no. 1). Key informants expressed that many students were supportive of the efforts, and students viewed tobacco use as having dangerous health consequences (quote no. 2). Additionally, students can support the efforts regardless of their academic or athletic backgrounds (quote no. 3). 

Students who served as campus leaders, student senate members, student health advisory committee members, or peer health educators played a crucial role in student engagement in tobacco-free efforts in CCCs, as they are respected by faculty and other leaders on campus (quote no. 4). Informants felt that it is important to educate students and staff to bring awareness to why a TFP is essential and beneficial (quote no. 5).

Key informants reported that hiring paid interns is an excellent way of getting students involved in TFP efforts and that colleges with paid and trained interns yielded better commitment and quality of work. According to one key informant from a community-based organization, the most helpful way to push the policy forward is to use students’ voices, whether in education or advocacy, and the best way to achieve that is through paid student internships (quote no. 6). A college administrator also expressed that student interns enhanced both themselves and the policy work (quote no. 7).

### Theme 2: Myriad levels of student engagement in tobacco-policy work

The second theme describes the concrete tasks in which the students partake in TFP efforts. These efforts are categorized into information gathering, education and awareness, advocacy, and activism. Data collection, observational studies, surveys, and focus groups are examples of information-gathering activities. Health fairs, presentations, and tabling are examples of activities that promote education and awareness. Examples of advocacy activities for TFPs included generating peer support, being actively involved in meetings, creating videos, testifying at stakeholder or college board meetings, and participating in the student health advisory committee. Activism in TFPs can be participation in rallies, garnering letters of support from student clubs, picking up cigarette butts, and performing park clean-ups. As one key informant mentioned, involving students in TFPs is vital (quote no. 8). Similarly, by partaking in different activities, students can build support from other decision-making bodies.

The range of student engagement in tobacco control policy work also allows students to bring their own creativity to these efforts, such as with artwork or videos that use different mediums to highlight policy efforts (quote nos. 9 and 10). Key informants highlighted that students could either lead tobacco-control efforts or take a supporting role. One key informant described how students took ownership (quote no. 11). However, according to another key informant, efforts on their campus involved students in a less active, but still important, role (quote no. 12). Once students are in the space of tobacco control policy work, they are likely to become advocates for broader tobacco control efforts (quote no. 13). Finally, one key informant described the benefits of using the Truth Initiative grant funding to hire 1 to 2 students (quote no. 14). For community college students, compensation for participation was important.

### Theme 3: Benefits of student engagement

The third theme describes the benefits of student engagement and the influence of students on the policy journey. A student services coordinator at one college best exemplified this theme (quote no. 15) by emphasizing the value of putting students in leadership positions. Three subthemes emerged on further analysis: 1) student influence on college decision-making communities or leaders, 2) student impact on policy, and 3) student skill-building and education.

Students influenced multiple groups, the first of which was faculty and staff, as they care about what students want on campus (quote no. 16). Moreover, students also influenced the board of trustees, a key community college governing body, to approve a TFP by providing evidence of student support on campus (quote no. 17). Given the shared governance of the CCC system, decision makers valued the support of students. Lastly, when a group of students is involved, they often attract other students to join advocacy efforts. For example, one college that has a strong collegiate athletics program worked with its student body president to bring the entire sports team to their tobacco-free campus events.

Second, students affect policy by bringing unique perspectives, roles, representations, and life experiences. One external community partner described just how extensive this impact was: what started with a paid internship ultimately led to the passing of a student government resolution (quote no. 18). The impact was especially relevant for campuses that heavily involved student leaders, such as the student body president and student trustee (quote no. 19). Multiple key informants acknowledged that students valued social justice and equity as part of the policy efforts, especially more so than groups that were more concerned about individual freedom (quote no. 20). As another unique contribution, several key informants described narratives of students who smoked but were still supportive of a TFP and how they played a crucial role in policy messaging (quote no. 21). Similarly, a student with asthma brought another powerful narrative at council meetings and on campus where they spoke about how smoke irritated their lungs. Finally, students themselves benefited greatly from being involved in these tobacco control opportunities (quote no. 22). In addition to gaining experience, they also learned about the college policy process and gained a passion for tobacco control work (quote no. 23).

## Discussion

Establishing 100% tobacco-free community colleges is an effective strategy to reduce tobacco use (24,25). Given that the demographic profile of community college students tends to be young adults from communities of lower socioeconomic status and racial and ethnic minority families, a TFP could address tobacco-related health disparities (26). As of 2023, only 66% of California community colleges are 100% tobacco-free; therefore, it is a high priority for the remaining community colleges to adopt a TFP (3). In addition, given CCC’s shared governance structure in which students have a voice along with faculty and staff in college- and district-wide decision-making processes, student engagement is a key ingredient for policy. However, research on student engagement in college tobacco control policy is limited. Studies that have examined student engagement were often conducted in already 100% tobacco-free campuses and focused on the role of student engagement to improve TFP compliance (2,27,28). Findings showed that students report mixed feelings regarding their role and level of authority and often feel uncomfortable approaching others who are smoking on campus (2,27,28). The policy violators also expressed feeling uncomfortable being approached by student ambassadors; however, most of them reported the ambassadors approached them with kindness and they had a positive experience interacting with them (2). Nevertheless, to our knowledge, this is the first study that explores the roles of student involvement in TFP adoption efforts on community college campuses using a sample of 12 community colleges in California. Findings on how campuses leveraged student voices and involvement can serve as a roadmap for other colleges who are advocating for a TFP.

The first theme highlighted that many ways exist for students to get involved in TFP efforts, advocate for policy change, and ultimately achieve a tobacco-free campus. Students have some of the most effective voices to advocate for what they believe is right (29). Students do not need to come from any specific background to get involved in this work, as long as they are passionate and interested in campus involvement. They can become ambassadors or student interns who deliver presentations at classrooms or board meetings. Students can even informally support policy efforts by completing surveys, participating in tobacco-free events such as the Great American Smoke Out, and voicing their opinions about passing a TFP on their college campus. Community colleges could use a range of methods and channels for engaging students.

Students majoring in health-related disciplines are often the most deeply involved in tobacco-free efforts because they are the ones who have an interest in public health. Most students who lead tobacco control efforts on their campuses tend to major in health-related fields and have a passion to serve and improve community health (2,27,28). Administrators can reach out to students who are passionate about social justice and public health issues who can become advocates for TFP efforts. They could build advocacy skills, provide training, and create a space for students to lead these policy efforts. If successful in educating young adults about the negative impact of tobacco smoke, students from other fields or majors may be willing to participate in TFP efforts.

Lastly, community colleges should consider dedicated funds for student engagement positions, such as through internal campus funding or external grants like the Truth Initiative (https://truthinitiative.org/) that supports campus tobacco policy efforts. Having paid student interns is an effective way to engage students because they commit their time and energy to the work more than they would with a volunteer position. As Hunt and Scott noted, paid internships require interns to be more responsible and therefore provide much higher quality work (30). The large population of low-income students at community colleges (5) may be more likely to look for paid positions, and paid student internships would offer them the opportunity to earn money while building their work experience.

Theme 2 highlighted the myriad ways in which students can be actively involved once they enter the space of TFP work. They bring their creativity into the space, and as agents of change (31), students understand social norms around tobacco use among their peers in ways that are different from campus administrators and other professionals. Providing such an environment also makes participation more appealing and encourages students to develop passion and investment in tobacco policy work. For example, through the creation of artwork, students visually expressed themselves and demonstrated how a tobacco-free campus matters to them.

Additionally, college administrators and staff need to recognize that having students involved in TFPs creates an environment that is open to change since students can be champions of change. This aligns with the Centers for Disease Control and Prevention’s 2010 Best Practices User Guide, which stated, “Youth enhance state and local tobacco control efforts by challenging conventional thinking, advocating for policies, and changing the social norms around tobacco use” (14). However, college administrators and staff should keep in mind that the benefits of student engagement should outweigh the risks in tobacco control efforts, as one TFP compliance study found that students may not be the best to deliver the intervention (27).

This theme also emphasized that students’ level of involvement in TFPs mattered. This pattern highlighted the value of student engagement as students took ownership of TFP efforts on their campuses. This is an essential lesson that community colleges that are not yet tobacco-free can incorporate for more successful efforts. Lastly, involving students in policies at their school creates an avenue for them to get more involved in local and statewide tobacco-free policies, an excellent opportunity for training students on policy advocacy and tobacco control experience for the future.

The third theme captured the benefits of student engagement, as students influence other stakeholders, including faculty, staff, and the board of trustees. Students themselves also gain knowledge, experience, and passion for advocacy. The investment of students in showing support for policy results in faculty, staff, and decision makers being interested in moving policy forward because students really are the “consumers” of community colleges, a mindset that has had a positive impact on universities (32). Thus, engaging multiple student groups results in the policy gaining more traction. Each student who is engaged also brings in more students who can continue to expand the circle of student supporters as exemplified by the sports teams supporting advocacy in one community college.

The student viewpoint often focuses on issues that students are facing first-hand and are passionate about. This perspective places students in the forefront in gathering the student body’s support while representing the student voice. If students are not engaged, ensuring the student perspective can be easily forgotten. Because students are also most affected by policy changes, the personal stories they share can carry weight throughout the campus community, so providing a platform for them to speak is critical.

Being engaged in TFP advocacy does not send students home empty-handed, but rather offers them distinct hands-on opportunities as they grow into more informed and empowered individuals. This type of experiential learning is what the Association of American Colleges and Universities calls “high impact practices” that provide significant educational benefits for students who participate in them (33). In fact, emphasizing student advocacy engagement through movements like this is a major part of most colleges’ mission statements. An urban Bay Area campus aims to “inspire participatory global citizenship grounded in critical thinking and an engaged, forward-thinking student body.” Students can best grow in participatory citizenship when involved in advocacy work. Similarly, a larger Southern California urban campus’s goal was to “create conditions for empowerment, critical thinking, and informed civic engagement” for their students. Adopting a 100% TFP on campus is a prime example to foster this goal and to empower students and showcases how central to the college experience student engagement can be.

### Strengths and limitations

Although a multi-campus qualitative study provides a rich, nuanced lens to understanding student engagement efforts, our study has limitations. The semistructured interviews allowed respondents to discuss students’ involvement within the broader context of other barriers and facilitators of establishing campus TFPs. Among the 33 key informant interviews, 3 were students, which represented a small proportion. Identifying more students to participate as key informants may have shown a more in-depth perspective on their involvement, bringing in a greater volume of primary sources. This study team included 3 currently enrolled undergraduate students, all of whom were recent community college students themselves who were deeply involved in data collection, analysis, and writing of this manuscript; their engagement exemplifies yet another entry point to integrate student voices. Also, 4 campuses (2 with TFPs and 2 without) did not have student involvement in TFP efforts; nevertheless, we included them in this study, as key informants expressed difficulties in engaging community college students given their limited time on campus. Because the study was done with community college campuses and because of the small sample size (ie, 12 colleges), findings may not be generalizable to 4-year institutions or schools outside of California.

### Implications

Students are important partners in the journey to TFP adoption. As Jazwa et al noted, students are the most commonly cited contributors to advancing policy change (34). This is no coincidence. ACHA standards recommend a community-based approach to facilitate change; students, one of the most impactful groups in the community, must be engaged. Moreover, students can be involved and empowered in multiple ways through many doors and a range of activities. Students can be agents of change in leading community college policy efforts. Whether through internship programs, student government, or survey responses, the student voice has power that can advance community college TFPs. Considering the limited amount of research on student engagement in TFP adoption, this article highlights the key role of students in moving campuses toward comprehensive policies in the CCC system.

### Conclusions

Institutions of higher learning should leverage their campuses’ most important assets — students — and involve them in the full spectrum of interventions and advocacy. The themes described in this article emphasized not only multiple entry points for students’ involvement but that there is no “wrong door” to engage students. We recommend creating opportunities for students in a purposeful, intentional manner while being careful not to make them into tokens. Creating leadership opportunities for students can help to advance tobacco control opportunities and reduce tobacco-related disparities, especially in community colleges.
